# Estimation of irrigated crop artificial irrigation evapotranspiration in China

**DOI:** 10.1038/s41598-024-67042-5

**Published:** 2024-07-12

**Authors:** Han Gao, Jiahong Liu, Hao Wang, Chao Mei, Jia Wang

**Affiliations:** 1 College of New Energy and Environment, Jilin University, Changchun, 130021 China; 2 State Key Laboratory of Simulation and Regulation of Water Cycle in River Basin, China Institute of Water Resources and Hydropower Research, Beijing, 100038 China

**Keywords:** Irrigated crops, Actual evapotranspiration, Artificial irrigation evapotranspiration, Agricultural water management, China, Environmental social sciences, Hydrology

## Abstract

Agriculture water use accounts for 70% of the total water withdrawal worldwide. The evapotranspiration during crop growth is one of the important hydrological processes in the agricultural water cycle. This study proposed the concept of artificial irrigation evapotranspiration of irrigated crops to describe that the evapotranspiration caused by irrigation water use. Irrigated crops rely on two kinds of water sources: precipitation and irrigation water. With the construction of irrigation schemes, the artificial irrigation evapotranspiration plays an increasingly important role in the dualistic water cycle system of irrigated cropland. To reveal the amount of artificial irrigation evapotranspiration of 17 categories of irrigated crops in China, this study proposed a new quantitative model system which was established based on traditional evapotranspiration models and soil water balance models. Based on the new model system, we calculated the annual artificial irrigation evapotranspiration of irrigated crops for the period 2013 to 2017 in China. The results showed that the proportion of artificial irrigation evapotranspiration to the total evapotranspiration of irrigated crops was 41.3%, whose value was 228.1 km^3^ a^−1^. The artificial irrigation evapotranspiration in different agricultural water management regions were 90.0 km^3^ a^−1^ in the northeast region, 86.0 km^3^ a^−1^ in the southeast region, and relatively low 52.2 km^3^a^−1^ in the west region. The results of this study can provide methods for water management and policy–making in agricultural irrigated areas, and it can also provide a preliminary understanding of the influence of human activities on the dualistic water cycle in cropland.

## Introduction

Evapotranspiration (ET) is an important component of the surface hydrological cycle and energy exchange process^1^, which also provides a vital link of soil–plant-atmosphere-continuum (SPAC)^2,3^, it affects the process of precipitation redistribution in the region, as well as the water cycle and hydrological balance^4^. The natural hydrological cycle is gradually changing under the influence of land use dynamics and global climate change^5,6^. As irrigation is one of the direct human alterations of terrestrial water cycle^7^, the agricultural cropland water cycle gradually develops a “natural-social” dualistic water cycle system^8^, and an artificial collateral circulation of water “withdrawal- delivery-use-drainage- reuse” is formed in the natural water cycle, where farmers need to irrigate crop when the soil moisture is insufficient or drainage is required when the soil moisture is excessive or support effectiveness of leaching for salt management in case of arid and semi-arid conditions, in order to maintain a suitable moisture content for the normal growth of crops. Therefore, artificial irrigation and drainage have changed the conventional natural processes of runoff producing and confluence. The construction of irrigation and drainage projects and the improvement of water resource utilization and development have changed the natural water cycle way in cropland, which brought the changes of relationships among the replenishment, runoff, and discharge of cropland water resources. The process of irrigation-evapotranspiration has become a significant hydrological process, and human activities are the drivers on the social side of the dualistic water cycle^9^. Irrigation has been in the dominant position, especially in arid and semi-arid regions^7^, and the natural water cycle processes of runoff producing and confluence has played a minor role. The water circulation and transformation in irrigation districts will inevitably be influenced by artificial irrigation systems, and human activities have changed the hydrological processes which was dominated by precipitation and runoff mechanisms in irrigation districts.

Irrigation districts are productive primary for global food production but also large consumers of water^10^. Irrigated cropland only accounts for 20% of the total cultivated area, but it produces 40% of the total grain production, while rainfed land produces 60% of the grain production with 80% of the total cultivated area^11^.Agricultural irrigation water use accounts for approximately 70% of the total human water withdrawal^12,13^, and more than 40% of crops are produced under irrigation conditions^14,15^. Irrigation is an important tool for agricultural production^16^, which ensures food security. From 1999 to 2015, China’s cropland increased by 0.87%, and the demand for irrigation water increased by 1.97%^17^. In China, the irrigated cropland area exceeds 60 million hectares, accounting for 49% of the total cultivated land area, where more than 75% of the total grain yield as well as over 90% of the economic crop yield can be produced^18^. Irrigation increases crop transpiration and atmospheric water vapor content, and indirectly affects precipitation^19^. Meanwhile, irrigation will affect the climate conditions in various regions around the world^20^, especially hot extremes^21,22^. At present, irrigation water consumption accounts for a significant portion of water consumption in global socio-economic sections, especially in regions with developed irrigated agriculture but less precipitation. Vörösmarty et al.^23^ studied the impact of climate change on global agricultural systems and indicated that irrigated areas may significantly increase in the future. FAO also predicted that global irrigation water demand will increase by 10% by 2050^24^. Based on these circumstances, the increasing agricultural water demand will exacerbate the conflict between natural ecosystem consumptive water and artificial social system consumptive water especially agricultural irrigation water consumption. To alleviate this question, it is necessary to make more scientific water resource management policies. The research of irrigated water evaporation can directly reflect the level of impact of human irrigation activities on the natural-social dualistic water cycle, which has great significance for analyzing the influence of human activities on climate change and cropland water cycle. The main form of water consumption is evapotranspiration (ET), which is the link between global water, energy, and carbon cycling^25,26^. Studying evapotranspiration is crucial for exploring the role of the earth's surface in climate systems^27^. Evapotranspiration mainly includes soil evaporation and crop transpiration, or the water layer of flooded rice fields is also contributing to evaporation, which is not only a major component of water transport in agricultural ecosystems, but also a critical parameter for crop water use^28^. Accurately estimating crop evapotranspiration (crop water demand) can help the agricultural sections better manage and allocate water resources. During the process of crop growth, it is necessary to ensure sufficient water for crop normal development. Irrigation is an important measure to maintain crop production in arid and semi-arid areas^7,29^, and evapotranspiration is an important part of irrigation consumptive water, and only a small portion is lost due to water body evaporation, which belongs to non-consumption water which is rarely considered in the calculation^30^.

There are some researches about different components of evapotranspiration in cropland. Romaguera et al.^31^ used a deviation correction method to correct two types of evapotranspiration products, one of which was GLDAS's ET product, which was only driven by precipitation. Another ET product was based on remote sensing and included water evaporation for precipitation and irrigation. The difference in ET between the calibrated remote sensing product ET and GLDAS's ET product was the evapotranspiration caused by irrigation. Wu et al.^32^ had also used spatial analysis and aggregation methods, to quantify the global agricultural water appropriation with data derived from Earth observations, including global maps of rainfed and irrigated cropland evapotranspiration (ET), net water consumption due to irrigation, natural ET and so on.

This study proposed the concept of artificial irrigation evapotranspiration, which represented the portion of evapotranspiration caused by irrigation activities during the entire growing period of irrigated crops, and the concept of natural evapotranspiration which means the part of evapotranspiration caused by precipitation of irrigated crops. We analyzed and calculated artificial irrigation evapotranspiration of irrigated crops in different regions according to the division of agricultural water management regions in China published on FAO's Global Information System on Water and Agriculture-AQUASTAT. This study can inform the level of human influence on the dualistic water cycle system in agriculture, and promote the sustainable utilization and development of water resources in the agricultural section.

## Materials and methods

### Study area

**Figure 1:**
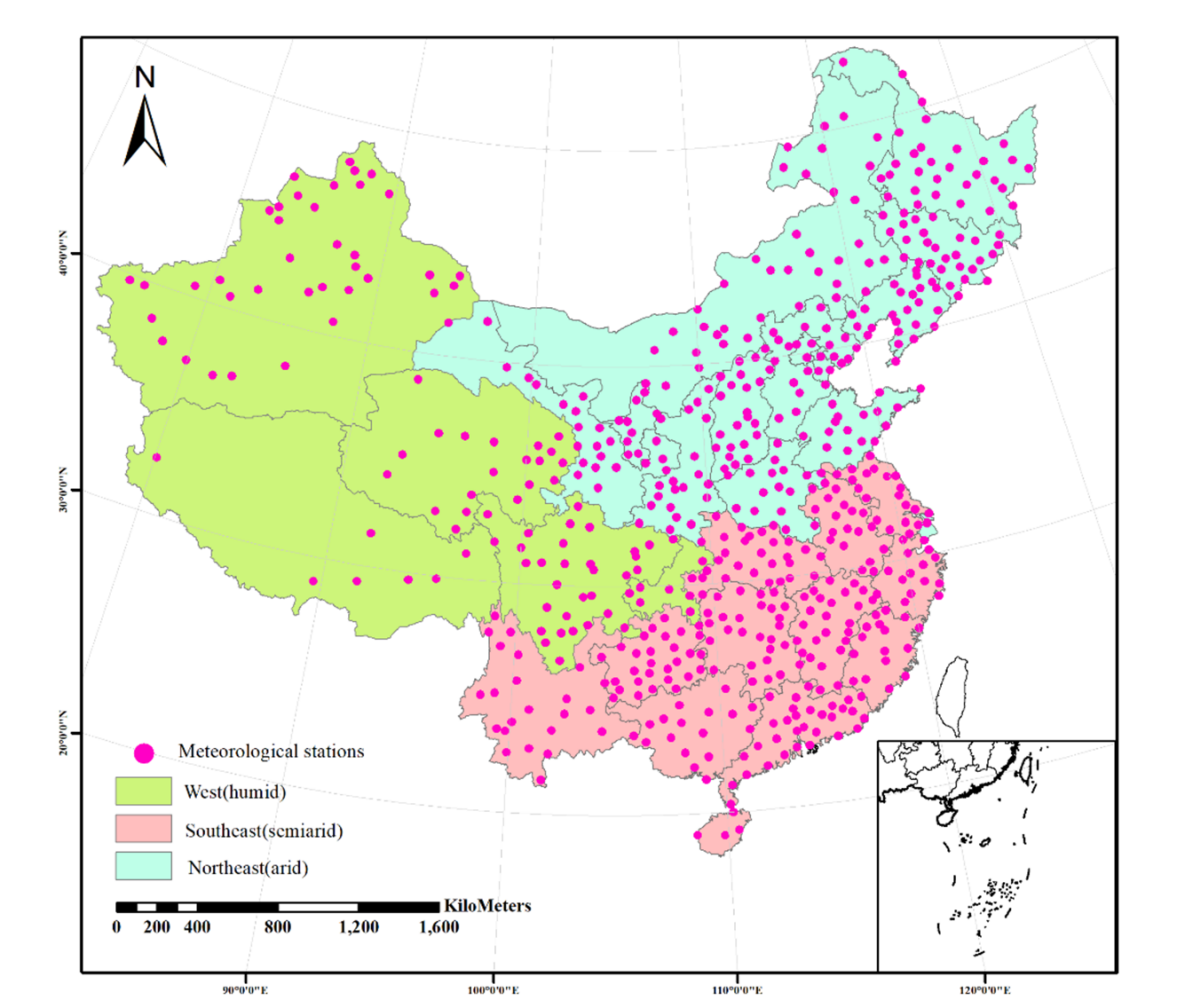
Distribution map of meteorological stations and agricultural water management regions in China. This figure was produced by using Arc Geographic Information System, ArcGIS, version 10.4.1 (https://www.esri.com/en-us/arcgis/products/arcgis-desktop/resources).

In this study, as shown in Fig. 1, China is divided into three regions referring to agricultural water management according to FAO: Northeast (NE)-arid, Southeast (SE)-semi arid and West(W)-humid (https://www.fao.org/aquastat/zh/data-analysis/irrig-water-use/irrigated-crop-calendars-Excel Format). The three regions contained provinces are shown in Table 1. Due to limitations in data availability, Taiwan Province was not considered in this calculation. The artificial irrigation evapotranspiration in different regions is calculated based on their corresponding agricultural irrigation water management conditions, then the results for China could be obtained.

**Table 1:** The crop harmonization of GAEZ and FAO; three regions contained provinces in China; irrigated crops sowing and harvesting time.

Crop		Northeast-Arid (Beijing , Tianjin, Gansu, Hebei, Heilongjiang, Henan, Inner Mongolia, Jilin, Liaoning, Ningxia, Shaanxi, Shandong, Shanghai, Shanxi)		Southeast-Semi arid (Anhui, Fujian, Guangdong, Guangxi, Guizhou, Hainan, Hubei, Hunan, Jiangsu, Jiangxi, Yunnan, Zhejiang)		West-Humid (Chongqing, Qinghai, Sichuan, Tibet, Xinjiang)	
GAEZ Name	FAO Name	Sowing	Harvesting	Sowing	Harvesting	Sowing	Harvesting
Wheat	Wheat	October	April	January	May	October	April
Rice	Rice	May	September	June	October	May	September
Maize	Maize	May	September	November	March	May	September
Other_cereals	Other cereals	May	September	November	March	May	September
Vegetables	Vegetables one	June	September	May	August	May	September
Crops_NES	Fruits	January	December	January	December	January	December
Soybean	Soybeans	May	September	April	August	May	September
Groundnut	Groundnuts	May	September	April	August	May	September
Rapeseed	Rapeseed	October	April	November	March	October	April
Sunflower	Sunflower	May	September	November	March	May	September
Potato_and_sweet_potato	Potatoes and other roots	May	September	April	August	May	September
Pulses	Pulses	May	September	April	August	May	September
Sugarcane	Sugarcane	January	December	January	December	January	December
Sugar beet	Sugar beet	May	October	April	August	May	October
Cotton	Cotton	May	November	April	October	May	November
Tobacco	Tobacco	April	August	April	August	April	August
Fodder_crop	Pasture permanent	January	December	January	December	January	December

### Data processing

As shown in Fig. 1, a total of 611 meteorological stations, with daily data for the period 2013–2017 were provided by the CHINA METEOROLOGICAL DATA SERVICE CENTRE (CMDC) of National Meteorological Information Centre (http://www.nmic.cn/). The dataset includes sunshine duration (h), wind speed (m·s^−1^), relative humidity (%), altitude (m), mean temperature (°C), minimum temperature (°C) as well as maximum temperature (°C), which were used to estimate reference crop evapotranspiration according to Penman–Monteith formula^33^. We summed the daily reference crop evapotranspiration data obtained from each station to monthly reference crop evapotranspiration data, and use Kriging interpolation method to obtain grid data. The datasets of precipitation, runoff, and soil moisture were from GLDAS Noah Land Surface Model L4 monthly 0.25 × 0.25 degree V2.1 (GLDAS_NOAH025_M) in 2013–2017, which were used to estimate natural evapotranspiration of irrigated crops. The soil moisture are available for four soil layers (0–10 cm, 10–40 cm, 40–100 cm, 100–200 cm)^34^. We used data on field capacity to calculate soil water stress factor-k_s_ from the related achievements^35^. The distribution dataset of irrigation crops in China was from Global Agro-Ecological Zones (GAEZ + 2015 Annual Crop Data), and it provided the global distribution as well as harvested area of 26 rainfed and irrigated crops in 2015^36,37^. We extracted and sorted out the irrigation crops using China’s administrative boundaries, resulting in 17 categories of irrigated crops, as shown in Table 1. The crops calendar including the months of sowing and harvesting in different regions was sourced from FAO-AQUASTAT statistical data, (https://www.fao.org/aquastat/zh/data-analysis/irrig-water-use/irrigated-crop-calendars-Excel Format). The crop harmonization of GAEZ and FAO was shown in Table 1, The crop coefficient k_c_ was sourced from the FAO-AQUASTAT database, as shown in Table S1 ( https://www.fao.org/aquastat/zh/data-analysis/irrig-water-use). All datasets re-gridded to the 5′ by 5′ resolution based on area-averaging^38^.

### Estimation model of artificial water evaporation of irrigated crops

**Figure 2:**
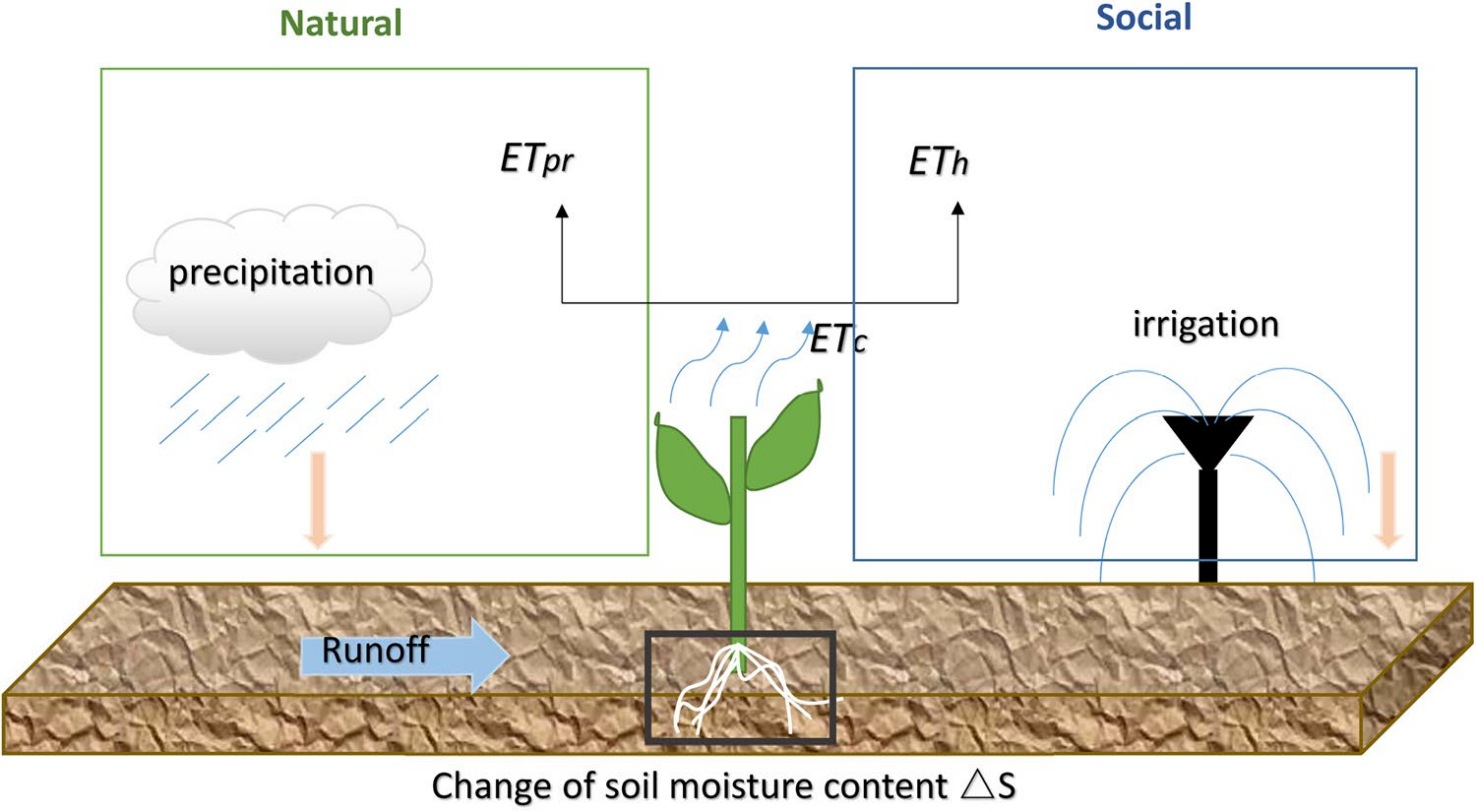
Water balance diagram for irrigated crops.

In irrigated cropland, when precipitation cannot effectively meet the needs of crop growth due to the mismatch between the spatiotemporal distribution of natural precipitation and the water demand of irrigated crops, so artificial allocation of water resources is needed. It means that there are two sources of water in irrigated cropland: precipitation and irrigation. Therefore, according to the water sources, the evapotranspiration of irrigated cropland can be divided into two categories: natural evapotranspiration (caused by precipitation) and social evapotranspiration (caused by irrigation) which is the same as artificial irrigation evapotranspiration for irrigated agriculture. The sum of the two is the actual evapotranspiration of irrigated crops throughout the whole growing period, which is the total evapotranspiration of a specific crop. A water balance diagram for irrigated crop based on root zone was established, as shown in Fig. 2.

The calculation formula for the actual evapotranspiration ($ET_{c}^{i}$) of irrigated crop *i* throughout the whole growth period is:

1 $$ET_{c}^{i} = k_{s}^{i} \times k_{c}^{i} \times ET_{0}$$

$k_{c}^{i}$ is the crop coefficient of irrigated crop *i*, which depending on the stages of crops growth of irrigated crop *i*,* i* represents the crop code. This study gave four stages of crop growth (initial stage, development stage, middle stage, and end stage) of the crops, provided by FAO-AQUASTAT and Siebert and Döll’s^4^research, as shown in Table S1. Referring to the processing method of crop coefficients in Huang et al.'s^39^ study, the equivalent $k_{c}^{i}$ values for each month during crop growth were recalculated, and the calculation results are shown in Table S2. $k_{s}^{i}$ is the soil water stress factor of irrigated crop *i*. When the crop is with sufficient water supply during the growth, *k*_*s*_ = 1, when irrigation is limited by water supply conditions and cannot meet the water demand of the crop, it is necessary to modify the evapotranspiration calculation of the crop under non-sufficient water supply conditions to obtain the actual evapotranspiration of the crop. This study used the Campbell method to calculate $k_{s}^{i}$, the formula is as follows:

2 $$k_{s}^{i} = \frac{2}{{1 + \left( {\frac{{\theta^{i} }}{{\theta_{f} }}} \right)^{ - 2} }}$$

$\theta^{i}$ is the weighted average of soil moisture in each layer, $mm^{3} /mm^{3}$,which depending on the effective root zone of irrigated crop i; $\theta_{f}$ is the field capacity, $mm^{3} /mm^{3}$; When $k_{s}$ > 1, take 1. The actual evapotranspiration of irrigated cropland may be limited by water supply conditions, therefore, this study assumed that irrigated crops growing in humid regions cannot be subjected to water stress during their growth, and irrigated crops growing in arid and semi-arid regions may be subjected to water stress during their growth due to water scarcity and climate conditions.

A complete crop evapotranspiration model used the Penman–Monteith formula to calculate the reference crop evapotranspiration ($ET_{0}$) has been established by FAO^39^.

3 $$ET_{0} = \frac{{0.408\Delta {(}R_{n} - {\text{G) + }}\gamma \frac{900}{{T + 273}}U_{2} \left( {e_{s} - e_{a} } \right)}}{{\Delta + \gamma \left( {1 + 0.34U_{2} } \right)}}$$

$ET_{0}$ is reference crop evapotranspiration, mm d^−1^; $\Delta$ is slope vapor pressure curve, kPa°C^−1^; $R_{n}$ is net radiation at the crop surface, MJ m^−2^ d^−1^; ${\text{G}}$ is soil heat flux density, MJ m^−2^ d^−1^; ${\upgamma }$ is psychrometric constant, kPa℃^−1^; $T$ is air temperature at 2 m height, °C; $U_{2}$ is wind speed at 2 m height, m s^−1^; $e_{s}$ is saturation vapor pressure, kPa; $e_{a}$ is actual vapor pressure, kPa. The factors affect the reference crop evapotranspiration mainly include radiation, temperature, wind speed, and saturation water vapor pressure difference. Radiation can be calculated by sunshine time, while saturation water vapor pressure difference can be calculated by temperature and relative humidity^39^.

This study calculated the artificial irrigation evapotranspiration $ET_{h}$ based on the difference between the actual crop evapotranspiration and the natural evapotranspiration of cropland:

4 $$ET_{h}^{i} = ET_{c}^{i} - ET_{pr}^{i}$$

$ET_{h}^{i}$ is the artificial irrigation evapotranspiration of irrigated crop *i* , $ET_{pr}^{i}$ is the natural evapotranspiration of irrigated crop *i* , which can be calculated based on the soil moisture balance formula:

5 $$ET_{pr}^{i} = P^{i} - R^{i} - \Delta S^{i}$$

$P^{i}$ is the precipitation during the growing periods of irrigated crop *i* , mm; $R^{i}$ is the runoff during the growing periods of irrigated crop *i*, mm; $\vartriangle S^{i}$ is the change of the soil moisture content within the effective root zone of irrigated crop *i*, mm.

Here, the proportion of human involvement in different irrigated crops growth processing is calculated as follows:

6 $$\alpha^{i} = \frac{{ET_{h}^{i} }}{{ET_{c}^{i} }}$$

where $\alpha^{i}$ is the artificial irrigation evapotranspiration proportion of actual evapotranspiration of crop *i*. The larger the value is, the higher the level of human involvement is.

According to the growing calendar of irrigated crops in China released by FAO, it can be obtained that the same crop may have different sowing and harvesting times in different regions, which was affected by various agricultural department management polices, climate conditions^32^, etc.. And there is a phenomenon of crop growth straddling the old and new years, for example, winter wheat in China was sown in the winter of this year and would be harvested in the next year. Therefore, for the convenience of calculation and analysis, this study gave the following assumption: the evapotranspiration during the growth period belonged to the year when the crop was sown. The planting calendar of crops did not change with climate change, and the groundwater in all regions where irrigated crops were cultivated was in a dynamic equilibrium state over the years.

## Results and discussion

**Figure 3:**
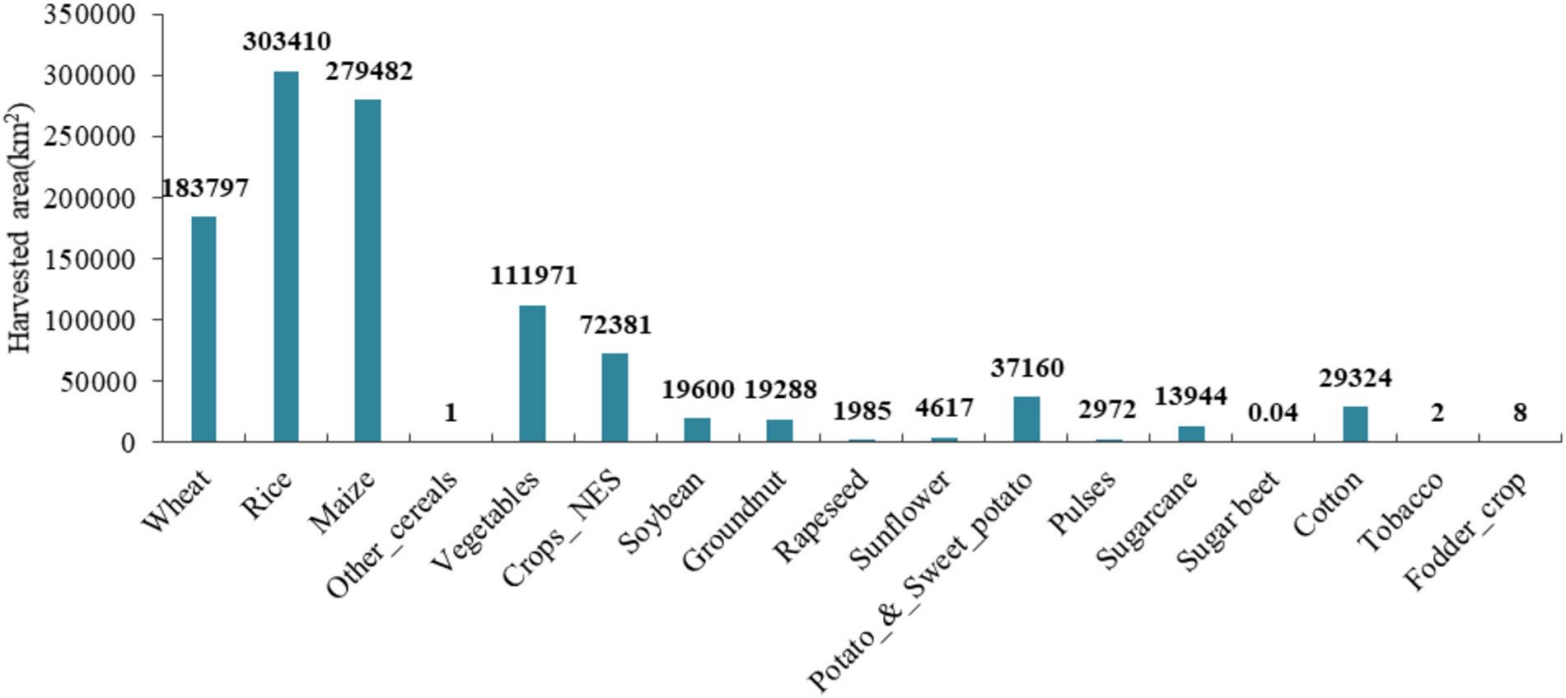
The harvested area (km^2^) for all irrigated crops in China.

**Figure 4:**
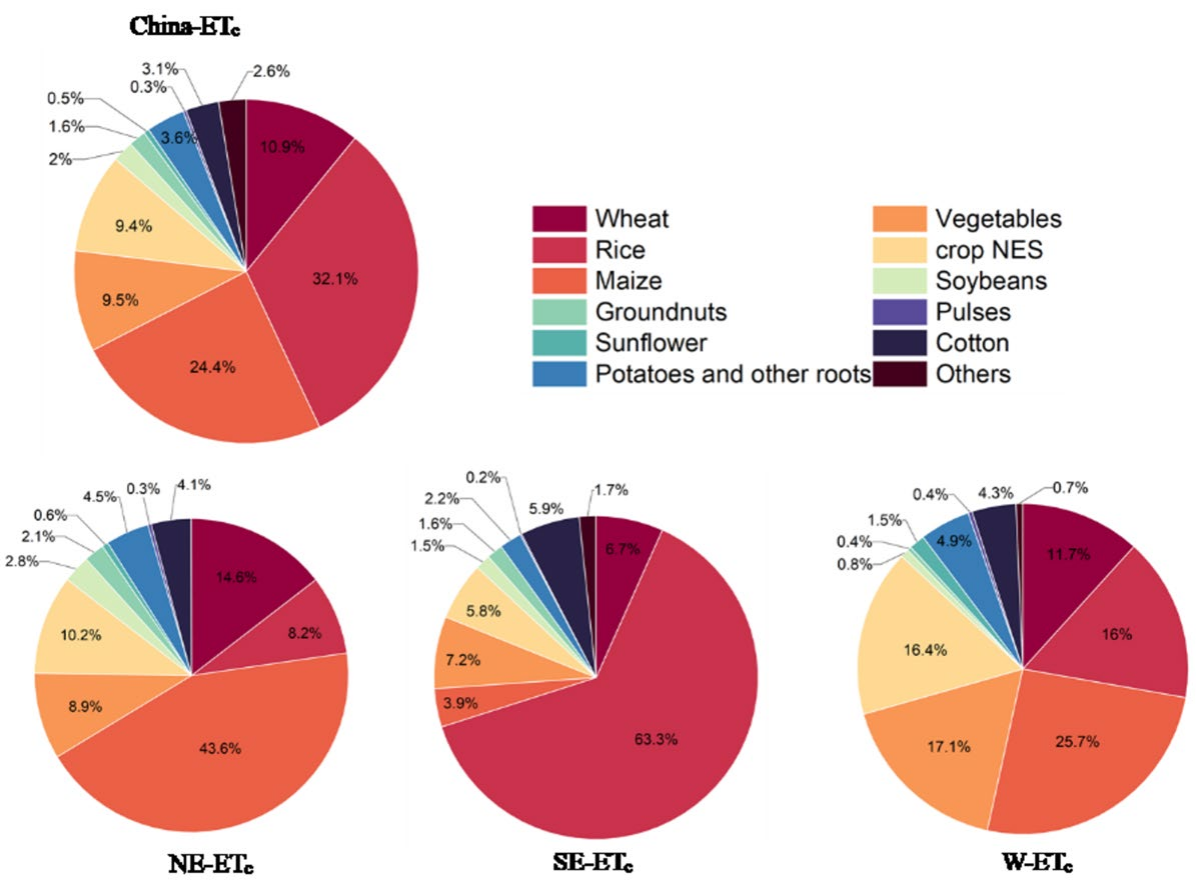
The contribution rate of each irrigated crops’ ET_c_ to the total ET_c_ in China and each region.

In this study, we calculated actual crop evapotranspiration for 17 categories of irrigated crops in China for the period 2013–2017.The total ET_c_ was 552.2 km^3^a^−1^ in the crop growing period. The ET_c_ for all irrigated crops were shown in Table 2. In China, the largest ET_c_ was calculated for the rice (177.2 km^3^a^−1^), maize (134.9 km^3^a^−1^), wheat (60.1 km^3^a^−1^), vegetables (52.5 km^3^a^−1^). The rather high amounts can be explained by the huge crop harvested area in China (see Fig. 3). In 2015, the total harvested area of irrigated crops was 1,079,941 km^2^. The harvested area of irrigated crops in the Northeast, Southeast and West regions were respectively 479,545 km^2^, 451,830 km^2^ and 148,566 km^2^. The Northeast and Southeast regions are the major regions for agricultural cultivation and development, most of the crops are planted in these two regions, therefore the irrigated crop evapotranspiration in both regions was also higher than that of west region. Rice, maize and wheat were the primary grain crops in China, their harvested areas were 303,410 km^2^, 279,482 km^2^ and 183,797 km^2^, respectively. Rice had the largest proportion 32.1% of total irrigated crops evapotranspiration. Besides, maize (24.4%), wheat (10.9%), vegetables (9.5%), Crops_NES (9.4%) also accounted for relatively large proportions of total irrigated actual crop evapotranspiration. The proportion of various irrigated crop evapotranspiration to the total actual evapotranspiration value of each region is shown in the Fig. 4. The irrigated crop actual evapotranspiration in NE and SE regions of China accounted for a largest proportion (84%) of the total irrigated crop actual evapotranspiration, while that in west region was only 16%. The ET_c_ of Other_cereals (238,880 m^3^a^−1^), sugar beet (19,592 m^3^a^−1^), tobacco (1,399,948 m^3^a^−1^) and Fodder_crop (3,644,748 m^3^a^−1^) was fewer due to their small harvested area.

**Table 2:** Irrigated crop actual evapotranspiration (ET_c_) in different regions.

Crop	ET_c_(million m^3^a^−1^)			
	NE	SE	W	Total
Wheat	34,730	15,275	10,140	60,145
Rice	19,446	143,896	13,874	177,217
Maize	104,027	8749	22,192	134,969
Other_cereals	0.015	–	0.223	0.239
Vegetables	21,290	16,357	14,826	52,472
Crops_NES	24,394	13,204	14,153	51,751
Soybean	6729	3477	705	10,911
Groundnut	5047	3612	364	9023
Rapeseed	90	148	170	408
Sunflower	1515	–	1326	2842
Potato_and_sweet_potato	10,650	5020	4272	19,942
Pulses	834	363	336	1533
Sugarcane	–	13,451	397	13,847
Sugar beet	0.0196	–	–	0.0196
Cotton	9801	3631	3720	17,153
Tobacco	1.40	–	–	1.40
Fodder_crop	–	–	3.64	3.64
China	238,554	227,183	86,480	552,216

**Figure 5:**
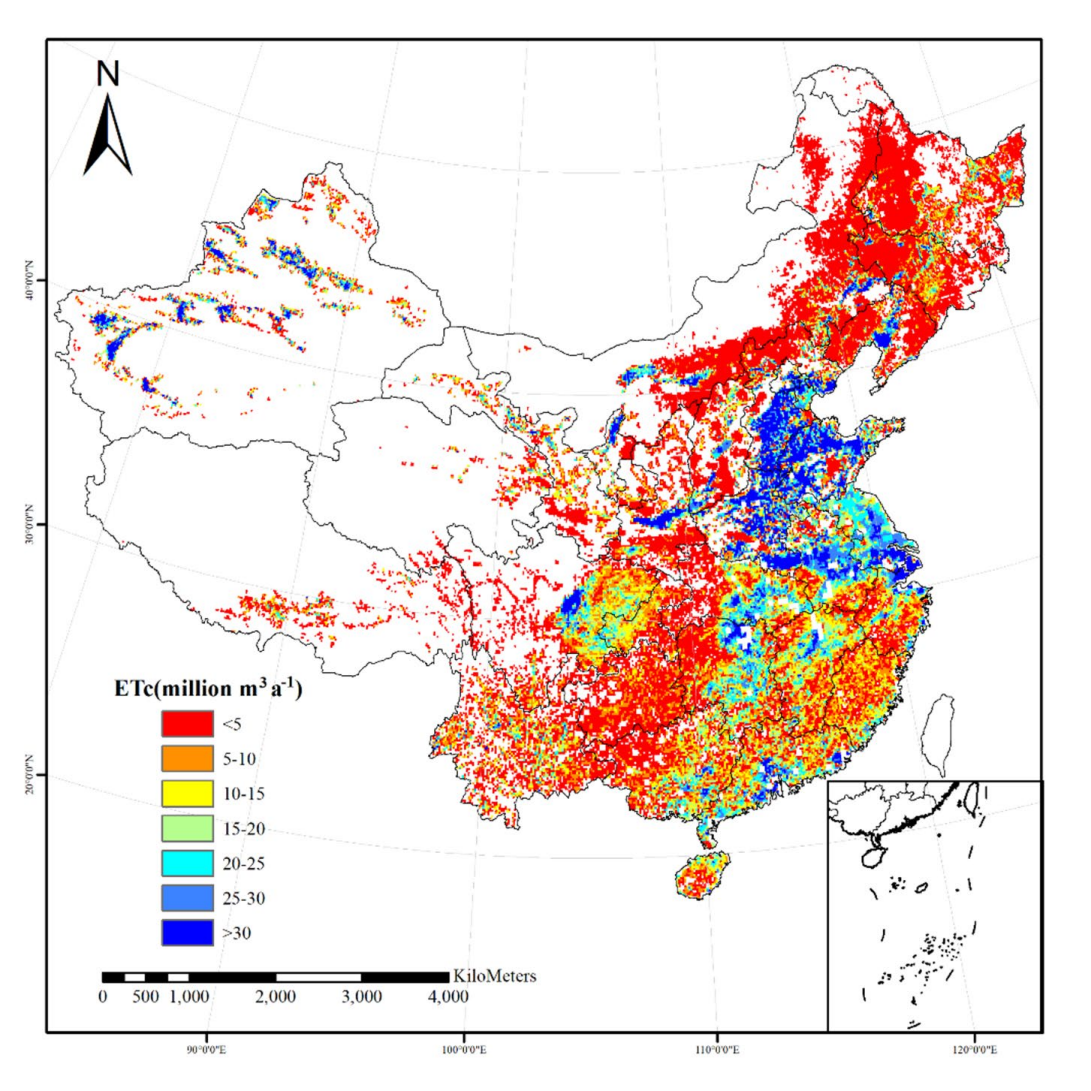
Spatial distribution map of irrigated crop actual evapotranspiration (ET_c_) in China. This figure was produced by using Arc Geographic Information System, ArcGIS, version 10.4.1 (https://www.esri.com/en-us/arcgis/products/arcgis-desktop/resources).

As shown in Fig. 5, eastern China features significant agricultural production and development areas characterized by relatively high crop evapotranspiration which is indicated by higher ET_c_ per grid cell, whereas fewer cells with high ET_c_ occur in Xinjiang province. Comparatively high crop evapotranspiration in the North China Plain, the Middle—Lower Valley of Yangtze River, Sichuan Basin and South China, reveal that these regions are the areas with most concentrated agricultural production both in terms of raising irrigated and intensifying rainfed crop production in China.

**Figure 6:**
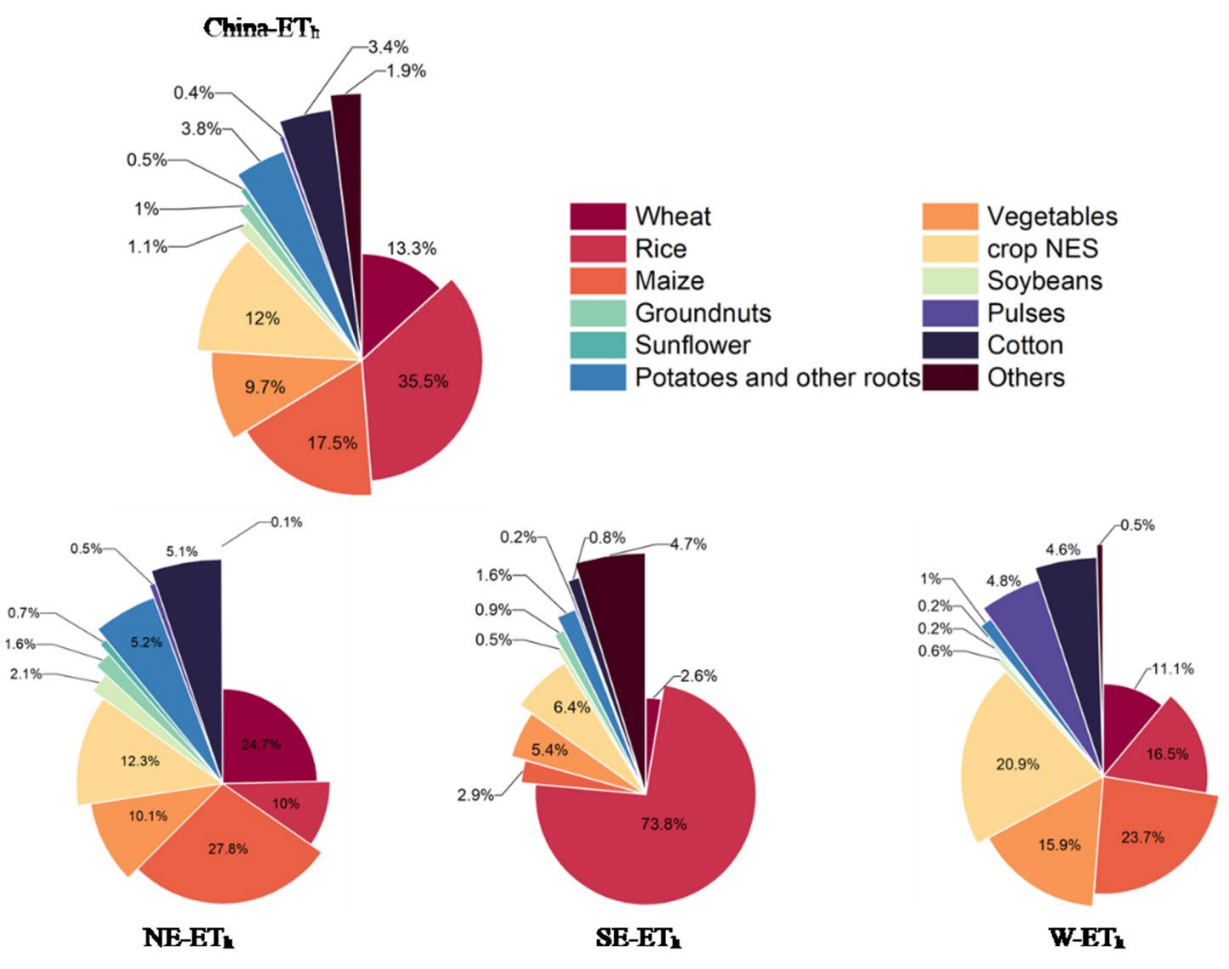
The contribution rate of irrigated crops’ ET_h_ to the total ET_h_ in China and each region.

**Figure 7:**
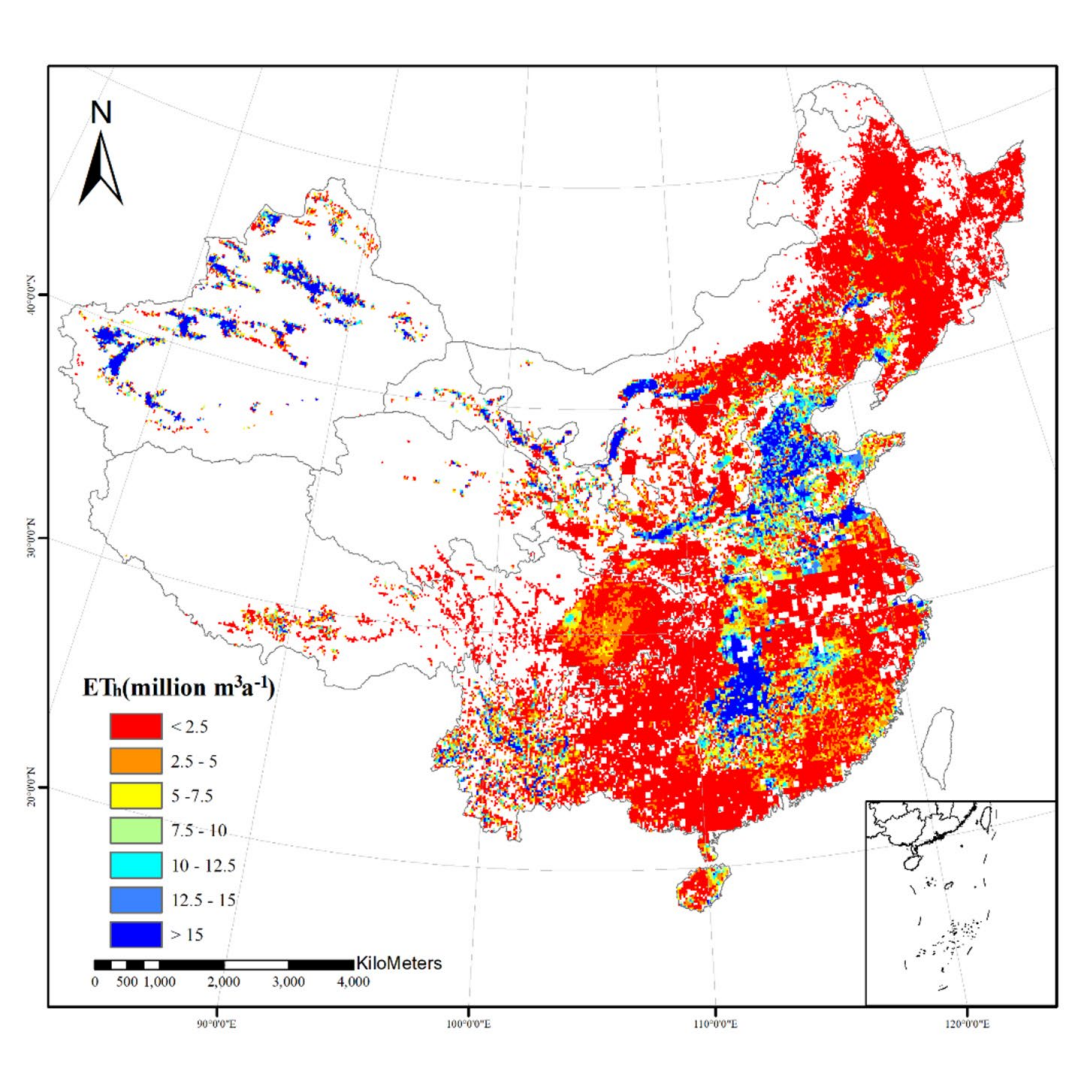
Spatial distribution map of artificial irrigation evapotranspiration (ET_h_) in China. This figure was produced by using Arc Geographic Information System, ArcGIS, version 10.4.1 (https://www.esri.com/en-us/arcgis/products/arcgis-desktop/resources).

Many water resource calculation models did not take into account the impact of human activities^40,41^. However, irrigation had a significant impact on grain prodution^42,43^, the irrigated areas are the principal drivers that increase irrigation water demand^44^. Therefore, human activities have significant influence on the sustainable development of irrigation agriculture and agricultural water use^45^. In this study ,the sources of water for irrigated cropland include natural precipitation and artificial irrigation water, and irrigated crop evapotranspiration can also be divided into natural evapotranspiration and artificial irrigation evapotranspiration based on different water sources. The total artificial irrigation evapotranspiration in China was 228.1 km^3^a^−1^. The values of ET_h_ were similar in the northeast and southeast regions, 90.0 km^3^a^-1^ and 85.6 km^3^a^−1^, respectively. The west region is relatively lower, 52.2 km^3^a^−1^. This was also because of the differences of harvested area in each region. The ET_h_ values of the main grain crops, wheat, rice, and maize, were 30.3 km^3^a^−1^, 81.0 km^3^a^−1^, and 40.0 km^3^a^−1^, respectively in China. The artificial irrigation evapotranspiration of these three crops accounted for 66.3% of the total artificial irrigation evapotranspiration in China, as shown in Fig. 6. In addition, the ET_h_ values of vegetables and Crops_NES were also relatively high, 22.0 km^3^a^−1^, 27.4 km^3^a^−1^, respectively. The ET_h_ proportion of these five crops reached 87.9%. The above 5 irrigated crops were the main producers of artificial irrigation evapotranspiration. According to the distribution of irrigated crops, rice was relatively less cultivated in the west and northeast regions. Most of the irrigated rice was cultivated in the southeast region, with a large harvested area of 251,052 km^2^. Therefore, the ET_h_ value of rice in the southeast region was the highest, 63.5 km^3^a^−1^. We found that although the harvested area of some irrigated crops in the northeast region was smaller than that in the southeast region, however, the ET_h_ values where were higher, such as wheat (22.2 km^3^a^−1^), maize (25.0 km^3^a^−1^), etc.. In three regions (NE,SE,W), every irrigated crop’s ET_h_ is displayed in Table 3. Spatial distribution map of artificial irrigation evapotranspiration in China is depicted in Fig. 7. This indicated that crops were also affected by the climate conditions of the cultivated area during their growth. The proportion of natural evapotranspiration would be relatively higher if there was higher precipitation, otherwise, the proportion of artificial irrigation evapotranspiration would be more.

**Table 3:** Artificial irrigation evapotranspiration (ET_h_) in different regions.

Crop	ET_h_(million m^3^a^−1^)			
	NE	SE	W	Total
Wheat	22,208	2260	5789	30,257
Rice	8962	63,450	8576	80,988
Maize	25,028	2514	12,336	39,877
Other_cereals	0.006	–	0.141	0.147
Vegetables	9073	4667	8298	22,038
Crops_NES	11,070	5487	10,872	27,428
Soybean	1854	435	313	2602
Groundnut	1411	789	109	2310
Rapeseed	50	62	97	208
Sunflower	594	–	542	1137
Potato_and_sweet_potato	4681	1370	2524	8575
Pulses	434	176	202	812
Sugarcane	–	4014	140	4154
Sugar beet	0.0108	–	–	0.0108
Cotton	4596	728	2394	7719
Tobacco	0.560	–	–	0.560
Fodder_crop	–	–	2.13	2.13
China	89,962	85,951	52,195	228,108

The $\alpha$ values of each crop in each region were shown in Table 4. Overall, the artificial irrigation evapotranspiration accounted for 0.413 of the total irrigated crop evapotranspiration which means that during the whole growth period of irrigated crops, there was 41.3% water that came from artificial irrigation in China. The $\alpha$ values were 0.377 in the northeast region, 0.378 in the southeast region, and 0.604 in the west region. Among all irrigated crops, the $\alpha$ values of wheat, sugar beet, pulses, rapeseed, Crops_ NES, Fodder_crop, and Other_cereals were greater than 50%, representing that artificial irrigation had a greater influence on the growth of these crops than precipitation. By analyzing the $\alpha$ values, it could be concluded that every irrigated crops’ $\alpha$ value generally followed this law: the $\alpha$ values in the Northeast and West regions were commonly higher than those in the Southeast region. In addition, we analyzed the agricultural water management regions provided by FAO and the spatial distribution characteristics of precipitation in China, it could be concluded that the annual precipitation decreased from southeast to northwest. For example, crop cultivation and growth mainly relied on artificial irrigation in Xinjiang Autonomous Region, Hetao Plain, Ningxia Plain. Furthermore, the rainy season started late, while ended early, which had a short duration in the northern regions. Therefore, some irrigated crops with higher water demand in the northeast region may rely on artificial irrigation during the early growing period.

**Table 4:** The level of human involvement in various irrigated crops growth processing in different regions.

Crop	$\alpha$			
	NE	SE	W	China
Wheat	0.639	0.148	0.571	0.503
Rice	0.461	0.441	0.618	0.457
Maize	0.241	0.287	0.556	0.295
Other_cereals	0.381	–	0.629	0.613
Vegetables	0.426	0.285	0.560	0.420
Crops_NES	0.454	0.416	0.512	0.530
Soybean	0.275	0.125	0.444	0.238
Groundnut	0.280	0.219	0.300	0.256
Rapeseed	0.552	0.417	0.569	0.510
Sunflower	0.392	–	0.409	0.400
Potato_and_sweet_potato	0.440	0.273	0.591	0.430
Pulses	0.521	0.485	0.602	0.530
Sugarcane	–	0.299	0.354	0.300
Sugar beet	0.550	–	–	0.550
Cotton	0.469	0.200	0.644	0.450
Tobacco	0.400	–	–	0.400
Fodder_crop	–	–	0.583	0.583

### Model validation

There have been some research results of ET_c_ about irrigated cropland in China. Most of these estimations were from global irrigated crop ET_c_ calculation results, and separately analyzed China as a significant agricultural country. Around the year 2000, the irrigated cropland area was 848,910 km^2^, and the ET_c_ for irrigated crops were 492.1 km^3^a^−1^ in China^40^. During the period 1998–2002, total irrigated crops ET_c_ were 429.9 km^3^a^−1^ as well as the irrigated harvested area was 905,740 km^2^ in eastern Asia^4^. In 2015, the irrigated area were 851,567 km^2^, the calculated total irrigated crops ET_c_ was 571.2 km^3^a^−132^. The results of crop evapotranspiration estimation depended on (i) the model used for quantification of the evapotranspiration and the procedures on determining crop coefficients and (ii) are under the influence of differences as well as limited precision regarding the input data and parameter configurations^41^. In this study, total irrigated cropland harvested area was 1,079,941 km^2^, and the ET_c_ for irrigated crop was 552.2 km^3^a^−1^.

There was no directly available statistical data for artificial irrigation evapotranspiration released in China. So we could only use indirect methods for verifying our results. According to the China Water Resources Bulletin in 2015 and 2015 Statistic Bulletin on China Water Actions, the agricultural water use was 385.1 km^3^ a^−1^, and the agricultural evapotranspiration rate was 64.3% (the evapotranspiration rate represents the ratio of total agricultural evapotranspiration to total water use), and the proportion of irrigated cropland area to the national irrigated area was 91.4% (this value is used to represent the proportion of agricultural water used for irrigated cropland). So the obtaining method of China’s agricultural irrigation evapotranspiration was that agricultural water use multiplied by evapotranspiration rate and the proportion of irrigated cropland area, which was 226.3 km^3^ a^−1^ in 2015. In this study, the estimated artificial irrigation evapotranspiration was 228.1 km^3^ a^−1^, which was very close to the statistical data, which indicated that the result of the calculation model was reasonable.

We collected and organized the irrigated cropland water use and the water evapotranspiration rate or agricultural irrigation evapotranspiration rate that was published in the Water Resources Bulletin of all provinces, then calculated and summarized the data from corresponding provinces included in the three agricultural water management regions, as shown in Table S3. The results showed that the total statistical agricultural irrigation evapotranspiration was 81.2 km^3^ a^−1^ in the Northeast region, 95.2 km^3^ a^−1^ in the Southeast region, and 50.0 km^3^ a^−1^ in the West region was in 2015. Based on the statistical data, it could be concluded that the calculation results of this study were relatively close to the statistical data. However, due to the following issues of the statistical data, there was still some slight differences between the statistical data and the estimated results in this study: (1) some statistical data lacked the evapotranspiration of agricultural irrigation in 2015, so this study used data from other years instead, such as Heilongjiang Province and Anhui Province (2) Water Resources Bulletin only provided total agricultural water use data or agricultural water consumption data (including agricultural irrigation water, forest and fruit land irrigation water, grassland irrigation water, fish pond replenishment water, and large-scale livestock and poultry breeding water) in the statistical data, which was larger than agricultural irrigation evapotranspiration, such as Guangdong Province, Hainan Province, etc. (3) some statistical data lacked agricultural evapotranspiration rate, and we could only use the national average instead to calculate, such as Fujian Province, and Shanghai City.

## Conclusions

In this paper, the definition of artificial irrigation evapotranspiration is clearly given, the process and mechanism of ET_h_ are analyzed, and the artificial irrigation evapotranspiration calculation model is established. The model was applied on the irrigated crops in China as an example and the results of the research enables to draw several main conclusions which are summarized as follows:

This paper identified the natural—social dualistic characteristic of irrigated crops which were cultivated in different agricultural water management regions, specially proposed the concept of artificial irrigation evapotranspiration and analyzed the evapotranspiration mechanism of various irrigated crops. The artificial irrigation evapotranspiration are regarded as the social evapotranspiration. The results reveal that the calculated contribution rates of artificial irrigation evapotranspiration of various irrigated crop in three agricultural water management regions of China are different. In 2015, the total irrigated crop actual evapotranspiration (ET_c_) in China was 552.2 km^3^ a^−1^, and the artificial irrigation evapotranspiration accounted for 41.3% of the total ET_c_ of irrigated crops, which was 228.1 km^3^ a^−1^. The total actual crop evapotranspiration and artificial irrigation evapotranspiration values in the southeast and northeast regions were similar, while the west region has the lowest value. The ET_c_ of the three main grain crops, wheat, rice, and corn, accounted for 67% of the total irrigated crop evapotranspiration in China. The proportion values of vegetables and Crops_NES, 9.5% and 9.4%, respectively, which were also higher than other irrigated crops. The maximum $\alpha$ value for other_cereals was 61%, followed by sugar beets (55%), pulses (53%), and Crops_NES (53%). This indicated that irrigation activities had various levels of impact on different crops during their growth process. The findings of the study on the impact of human irrigation activities on crop growth can promote better management of agricultural water resources, and have the potential to further rationalize the construction and management of irrigation infrastructure as well as to support food production for achieving food security, sustainable utilization and development of water resources in China in the future. Meanwhile, this study filled the gap in estimating artificial irrigation evapotranspiration of present research in China.

In this study, there are several uncertainty issues worth discussing and further considering: (1) Although the crop calendar provided in FAO gave corresponding sowing and harvesting time according to different agricultural water management regions, the real cultivating time may be slightly different due adaptation at some locations to regional climate, soil conditions and variety characteristics. Furthermore, mixed agriculture zones were not considered in this study due to data limitations. (2) We did not consider the irrigation modes for crops, such as sprinkler irrigation, micro irrigation, and film irrigation. This study only assumed that irrigate crops still accepted the traditional flood irrigation mode. (3) Furthermore, our study did not take into account the impact of irrigation on regional groundwater, this article assumed that groundwater was in a multi-year equilibrium state. In some regions, groundwater was extracted for irrigating crops, and excessive irrigation water could also leak and recharge groundwater (such as high water demand was needed in the early stage of rice growth). However, the above limitations did not bring about significant errors in the calculation results.

### Supplementary Information

Supplementary Tables.

## Data Availability

All data generated or analysed during this study are included in this published article [and its supplementary information files].
