# Supplementary material for: Estimation of irrigated crop artificial irrigation evapotranspiration in China
Source: Sci Rep. 2024 Jul 12;14:16142. doi: 10.1038/s41598-024-67042-5 (PMC11245542; doi:10.1038/s41598-024-67042-5)
Supplement: Supplementary file 1 — Supplementary Tables. [file 41598_2024_67042_MOESM1_ESM.docx]

Table S1 Crop coefficient k_c_, relative length of crop development stage, irrigated crop root depth

| **crop** | **crop coefficient(-)** | | | **Relative length of crop development stage (–)** | | | | **Root depth(m)** |
| --- | --- | --- | --- | --- | --- | --- | --- | --- |
|  | kcint | kcmid | kcend | Lint | Ldev | Lmid | Lend |  |
| Wheat | 0.4 | 1.15 | 0.3 | 0.15 | 0.25 | 0.4 | 0.2 | 1.25 |
| Rice | 1.2 | 1.1 | 0.8 | 0.17 | 0.18 | 0.44 | 0.21 | 0.5 |
| Maize | 0.3 | 1.2 | 0.6 | 0.17 | 0.28 | 0.33 | 0.22 | 1 |
| Other_cereals | 1 | 1 | 1 | 0.1 | 0.6 | 0.2 | 0.1 | 1.25 |
| Vegetables | 0.6 | 1.1 | 0.9 | 0.15 | 0.25 | 0.4 | 0.2 | 0.5 |
| Crops_ NES | 0.75 | 0.75 | 0.75 | 0.16 | 0.25 | 0.33 | 0.26 | 1 |
| Soybean | 0.4 | 1.15 | 0.5 | 0.15 | 0.2 | 0.45 | 0.2 | 0.6 |
| Groundnut | 0.4 | 1.15 | 0.6 | 0.22 | 0.28 | 0.3 | 0.2 | 0.5 |
| Rapeseed | 0.35 | 1.1 | 0.35 | 0.3 | 0.25 | 0.3 | 0.15 | 1 |
| Sunflower | 0.35 | 1.1 | 0.35 | 0.19 | 0.27 | 0.35 | 0.19 | 0.8 |
| Potato_and_sweet_potato | 0.5 | 1.15 | 0.75 | 0.2 | 0.25 | 0.35 | 0.2 | 0.4 |
| Pulses | 0.4 | 1.15 | 0.55 | 0.18 | 0.27 | 0.35 | 0.2 | 0.55 |
| Sugarcane | 1 | 1 | 1 | 0 | 0 | 1 | 0 | 1.2 |
| Sugar beet | 0.35 | 1.2 | 0.7 | 0.2 | 0.25 | 0.35 | 0.2 | 0.7 |
| Cotton | 0.35 | 1.2 | 0.6 | 0.17 | 0.33 | 0.25 | 0.25 | 1 |
| Tobacco | 0.5 | 1.2 | 0.8 | 0.15 | 0.25 | 0.4 | 0.2 | 1 |
| Fodder_crop | 1 | 1 | 1 | 0 | 0 | 1 | 0 | 1 |

Sources: kc (https://www.fao.org/aquastat/zh/data-analysis/irrig-water-use)

relative length of crop development stage, irrigated crop root depth(Siebert and Döll, 2010)

Table S2 Equivalent k_c_ value per month during crop growth stage in different agricultural water management regions

| crop | Equivalent k_c_ value per month during crop growth stage | | | | | | | | | | | | | | | | | | | | | |
| --- | --- | --- | --- | --- | --- | --- | --- | --- | --- | --- | --- | --- | --- | --- | --- | --- | --- | --- | --- | --- | --- | --- |
|  | Northeast | | | | | | | | Southeast | | | | | | | West | | | | | | |
| Wheat | | 0.40 | 0.59 | 1.01 | 1.15 | 1.15 | 1.13 | 0.90 | 0.42 | 0.85 | 1.15 | 1.15 | 0.73 |  |  | 0.40 | 0.59 | 1.01 | 1.15 | 1.15 | 1.13 | 0.90 |
| Rice | | 1.19 | 1.13 | 1.10 | 1.10 | 0.95 |  |  | 1.19 | 1.13 | 1.10 | 1.10 | 0.95 |  |  | 1.19 | 1.13 | 1.10 | 1.10 | 0.95 |  |  |
| Maize | | 0.31 | 0.72 | 1.18 | 1.20 | 0.76 |  |  | 0.31 | 0.72 | 1.18 | 1.20 | 0.76 |  |  | 0.31 | 0.72 | 1.18 | 1.20 | 0.76 |  |  |
| Other_cereals | | 1.00 |  |  |  |  |  |  | 1.00 |  |  |  |  |  |  | 1.00 |  |  |  |  |  |  |
| Vegetables | | 0.64 | 1.01 | 1.10 | 0.90 |  |  |  | 0.64 | 1.01 | 1.10 | 0.90 |  |  |  | 0.61 | 0.90 | 1.10 | 1.10 | 1.00 |  |  |
| Crops_ NES | | 0.75 |  |  |  |  |  |  | 0.75 |  |  |  |  |  |  | 0.75 |  |  |  |  |  |  |
| Soybean | | 0.42 | 0.94 | 1.15 | 1.50 | 0.78 |  |  | 0.42 | 0.94 | 1.15 | 1.50 | 0.78 |  |  | 0.42 | 0.94 | 1.15 | 1.50 | 0.78 |  |  |
| Groundnut | | 0.40 | 0.62 | 1.11 | 1.15 | 0.88 |  |  | 0.40 | 0.62 | 1.11 | 1.15 | 0.88 |  |  | 0.40 | 0.62 | 1.11 | 1.15 | 0.88 |  |  |
| Rapeseed | | 0.35 | 0.35 | 0.52 | 0.97 | 1.10 | 1.10 | 0.73 | 0.35 | 0.43 | 0.93 | 1.10 | 0.82 |  |  | 0.35 | 0.35 | 0.52 | 0.97 | 1.10 | 1.10 | 0.73 |
| Sunflower | | 0.35 | 0.68 | 1.07 | 1.10 | 0.73 |  |  | 0.35 | 0.68 | 1.07 | 1.10 | 0.73 |  |  | 0.35 | 0.68 | 1.07 | 1.10 | 0.73 |  |  |
| Potato_and_sweet_potato | | 0.50 | 0.76 | 1.13 | 1.15 | 0.83 |  |  | 0.50 | 0.76 | 1.13 | 1.15 | 0.83 |  |  | 0.50 | 0.76 | 1.13 | 1.15 | 0.83 |  |  |
| Pulses | | 0.40 | 0.73 | 1.13 | 1.15 | 0.78 |  |  | 0.40 | 0.73 | 1.13 | 1.15 | 0.78 |  |  | 0.40 | 0.73 | 1.13 | 1.15 | 0.78 |  |  |
| Sugarcane | | 1.00 |  |  |  |  |  |  | 1.00 |  |  |  |  |  |  | 1.00 |  |  |  |  |  |  |
| Sugar beet | | 0.35 | 0.67 | 1.18 | 1.20 | 1.19 | 0.88 |  | 0.35 | 0.69 | 1.18 | 1.20 | 0.95 |  |  | 0.35 | 0.67 | 1.18 | 1.20 | 1.19 | 0.88 |  |
| Cotton | | 0.35 | 0.47 | 0.83 | 1.15 | 1.20 | 1.06 | 0.59 | 0.35 | 0.47 | 0.83 | 1.15 | 1.20 | 1.06 | 0.59 | 0.35 | 0.47 | 0.83 | 1.15 | 1.20 | 1.06 | 0.59 |
| Tobacco | | 0.52 | 0.92 | 1.20 | 1.20 | 0.85 |  |  | 0.52 | 0.92 | 1.20 | 1.20 | 0.85 |  |  | 0.52 | 0.92 | 1.20 | 1.20 | 0.85 |  |  |
| Fodder_crop | | 1.00 |  |  |  |  |  |  | 1.00 |  |  |  |  |  |  | 1.00 |  |  |  |  |  |  |

Table S3 Statistical data of each province in different regions (symbol “/” represents the statistical data of some province directly provides the artificial irrigation evapotranspiration)

| **NE-Province** | irrigated cropland water use(km^3^) | | the water evaporation rate(-) | agricultural irrigation evapotranspiration (km^3^) |
| --- | --- | --- | --- | --- |
| Beijing | 0.38 | 0.71 | | 0.27 |
| Tianjin | 0.93 | 0.88 | | 0.81 |
| Gansu | 8.74 | 0.72 | | 6.26 |
| Hebei | 12.4 | 0.74 | | 9.16 |
| Heilongjiang | 29.6 | 0.65 | | 19.4 |
| Henan | 11.1 | 0.67 | | 7.43 |
| Inner Mongolia | / | / | | 7.51 |
| Jilin | / | / | | 4.53 |
| Liaoning | / | / | | 5.92 |
| Ningxia | / | / | | 3.04 |
| Shaanxi | / | / | | 3.69 |
| Shandong | / | / | | 8.91 |
| Shanghai | 1.42 | 0.64 | | 0.92 |
| Shanxi | / | / | | 3.38 |
| ***Total*** |  |  | | **81.21** |

| **SE-Province** | irrigated cropland water use(km^3^) | the water evaporation rate(-) | agricultural irrigation evapotranspiration (km^3^) |
| --- | --- | --- | --- |
| Anhui | / | / | 9.63 |
| Fujian | 8.51 | 0.64 | 5.47 |
| Guangdong | / | / | 11.5 |
| Guangxi | / | / | 8.16 |
| Guizhou | / | 0.49 | 2.98 |
| Hainan | / |  | 1.67 |
| Hubei | 189.32 | 0.52 | 8.55 |
| Hunan | / | / | 9.81 |
| Jiangsu | / | / | 18.4 |
| Jiangxi | / | / | 7.24 |
| Yunnan | / | / | 6.98 |
| Zhejiang | / | / | 4.81 |
| ***Total*** |  |  | **95.20** |

| **W-Province** | irrigated cropland water use(km^3^) | the water evaporation rate(-) | agricultural irrigation evapotranspiration (km^3^) |
| --- | --- | --- | --- |
| Chongqing | 2.59 | 0.54 | 1.40 |
| Qinghai | / | / | 0.87 |
| Sichuan | / | / | 7.25 |
| Tibet | / | / | 11.77 |
| Xinjiang | / | / | 28.66 |
| ***Total*** |  |  | **49.95** |
